# Preoperative measurement of femoral head using 3D-CT spherical fitting technique for the guidance of femoroplasty in arthroscopic treatment of cam-type femoroacetabular impingement: a cohort validation

**DOI:** 10.3389/fmed.2026.1732551

**Published:** 2026-09-23

**Authors:** Huichao Wang, HaoBo Liang, Yingjie Zhu, Kejie Fan, Youwen Liu, Ke Chen

**Affiliations:** 1 Department of Hip Joint Surgical Diagnosis and Treatment Center, Luoyang Orthopedic Traumatological Hospital, Luoyang, China; 2 Henan University of Chinese Medicine, Zhengzhou, China

**Keywords:** 3D-CT, cam deformity, femoroacetabular impingement, hip arthroscopy, spherical fitting technique

## Abstract

**Purpose:**

Arthroscopic femoroplasty is the primary treatment of Cam-type femoroacetabular impingement (FAI), in which the most important problem is how to evaluate Cam deformity sufficiently and remove osteophytes accurately. This study aimed to measure Cam deformity in each patient with 3D-CT spherical fitting technique, plan the resection range of Cam deformity preoperatively, so as to achieve precise resection intraoperatively and evaluate the prognosis.

**Methods:**

Patients of Cam-type FAI and underwent hip arthroscopy during August 2024 to February 2025 were involved. Preoperatively, all patients underwent CT scan, the spherical fitting measurement was performed to plan the resection range of Cam deformity, and the surgical operation was guided according to the plan. The post-operative alpha angle, femoral head-neck off-set ratio, as well as the mHHS and VAS at 3 and 6 months after the surgery were collected, and compared with that before the surgery.

**Results:**

29 patients were included and followed with no missing cases. The postoperative average alpha angle was 44.2 ± 4.1 degrees, significantly lower than that before surgery (75.3 ± 6.3) (*p* = 0.017). The postoperative average femoral head-neck off-set ratio was 0.20 ± 0.04, significantly better than that before surgery (0.16 ± 0.06) (*p* = 0.033). The average mHHS increased sequentially at 3 and 6 months after surgery, which was 73.2 ± 14.5 (*p* = 0.015) and 77.9 ± 15.8 (*p* = 0.010) respectively, were higher than that before surgery (52.5 ± 11.7). The average VAS decreased sequentially from 3.4 ± 0.9 preoperatively to 2.4 ± 0.6 (*p* = 0.029) and 2.3 ± 0.8 (*p* = 0.025) at 3 and 6 months after surgery (*p* < 0.05).

**Conclusion:**

The 3D-CT spherical fitting technique combined with the outside-in hip arthroscopy approach provided promising short-term clinical and imaging outcomes for Cam-type FAI. This combined method may represent a reliable and reproducible strategy for achieving precise cam resection in hip arthroscopy.

## 1 Introduction

Femoroacetabular impingement (FAI) is a hip disorder predominantly affecting young adults and physically active individuals. Its clinical manifestations primarily include hip and groin pain, restricted joint mobility, and particularly pain during hip flexion (1, 2). Ganz et al. defined FAI as a pathological contact between the femoral head-neck junction and the acetabular rim during extreme hip motion, leading to damage of the labrum and surrounding cartilage (3). Based on the location of abnormal bony protrusions, FAI is classified into three types: Cam deformity: A bony protrusion at the anterosuperior femoral head-neck junction, which reduces the normal concave curvature of the femoral head-neck region, disrupts the spherical structure of the femoral head, and causes pathological impingement between the femoral head-neck and acetabular rim during hip flexion, resulting in labral and/or cartilage damage; Pincer deformity: An abnormally prominent acetabular rim that repeatedly collides with the femoral head-neck in movement, causing pain and restricted motion; Mixed deformity: A combination of both deformities (4).

Femoroplasty is the primary treatment for Cam-type FAI (5). This procedure involves resection of the Cam deformity to restore the normal spherical structure of the femoral head-neck junction and eliminate impingement (6). Ganz et al. first introduced the concept of Cam deformity and reported surgical hip dislocation to directly visualize and perform femoroplasty, restoring the femoral head's spherical structure (3, 7). With advancements in arthroscopy, hip arthroscopy has become the preferred treatment for symptomatic FAI, addressing Cam and Pincer deformities through femoroplasty and acetabuloplasty, respectively (8). However, unlike open surgery, hip arthroscopy faces challenges due to the narrow joint space, deep anatomical location, tight joint capsule, and lack of clear landmarks, limiting visualization and making it difficult to fully assess the extent of Cam deformity intraoperatively (9, 10). A systematic review of 348 hip arthroscopy revision cases found that residual impingement—due to residual mixed deformity (45%), residual Cam deformity (27%), or residual Pincer deformity (9%)—accounted for 81% of revision surgeries (11).

To address these challenges, thorough preoperative evaluation and precise planning of osteophyte resection are essential (12). While 3D-CT is widely used for preoperative planning due to its superior bone imaging compared to MRI (13, 14), it traditionally fails to clearly delineate Cam deformity boundaries, relying heavily on surgeon experience and leading to suboptimal resection accuracy (10, 15, 16). Marking Cam deformity boundaries on 3D-CT could significantly enhance preoperative planning. Some surgeons have reported intraoperative use of curved templates matched to femoral head size to identify Cam deformity margins (17–20). In this study, we proposed a spherical fitting technique to create a best-fit sphere based on each femoral head, which can visualize the Cam deformity in 3D-CT.

To improve surgical visualization, novel techniques have been explored. Traditional hip arthroscopy employs the Inside-out technique, where portals are established under traction and fluoroscopic guidance using the Seldinger percutaneous method, followed by capsulotomy (21, 22). Recently, the Outside-in hip arthroscopy technique has been introduced. This approach involves creating an extracapsular space anteriorly, locating the femoral neck, and entering the joint capsule without traction or fluoroscopy (23–25).

Based on these innovations, this study aimed to integrate 3D-CT spherical fitting for preoperative Cam deformity assessment and resection planning with the Outside-in arthroscopy technique to achieve enhanced visualization, precise intraoperative Cam localization, and complete resection. The clinical outcomes of this combined approach are systematically evaluated.

This study aimed to conduct personalized evaluation of patients with Cam deformity using 3D-CT spherical fitting technique. The resection region of Cam deformity was planned, and postoperative imaging parameters and hip function scores were evaluated. Hypothesis: (1) With the help of preoperative planning using 3D-CT spherical fitting technique, the Cam deformity can be corrected postoperatively; (2) The patients had satisfied outcomes postoperatively; (3) The application of spherical fitting technique in preoperative planning is feasible and effective.

## 2 Methods

This study is a retrospective cohort analysis of patients diagnosed with Cam-type femoroacetabular impingement (FAI) who underwent hip arthroscopy at our institution between August 2024 to February 2025.

### 2.1 Inclusion and exclusion criteria

Inclusion Criteria: Presence of typical FAI symptoms and signs; Completion of CT and unilateral hip MRI within six months preoperatively, with all imaging performed at our institution; Alpha angle >55° on unilateral hip MRI (The alpha angle was measured from oblique coronal MRI.), confirming Cam-type FAI, and planned for hip arthroscopy at our institution; Surgical indications: persistent symptoms for ≥6 months despite conservative treatment, and MRI evidence of acetabular labral tear.

Exclusion Criteria: Revision arthroscopy; Radiographic evidence of osteoarthritis (Tönnis grade >1); History of peri-hip fracture, infection, or tumor; Autoimmune or systemic inflammatory diseases; Preoperative CT or MRI not performed at our institution.

### 2.2 Preoperative cam resection planning

All patients underwent preoperative 3D-CT scans using the 3D-CT spherical fitting technique for surgical planning. Pelvic CT Protocol: Patients were positioned supine on the CT table with neutral lower limb rotation. Standard axial CT images were acquired and exported as DICOM files to MIMICS 21.0 (Materialise, Belgium) for analysis.

3D Reconstruction: Using the “Advanced Segment” function in the “CT Bone” module, masks were created for each bone with the following parameters: Sensitivity: 70%, Threshold: 226-2222 HU, Gap Closing Distance: 3 pixels, “Fill Long Bones” option enabled. The “Calculate Part” function generated 3D models of the pelvis and proximal femur. Manual optimizations (mask expansion, void filling, surface smoothing) were applied (Figure 1).

**Figure 1:**
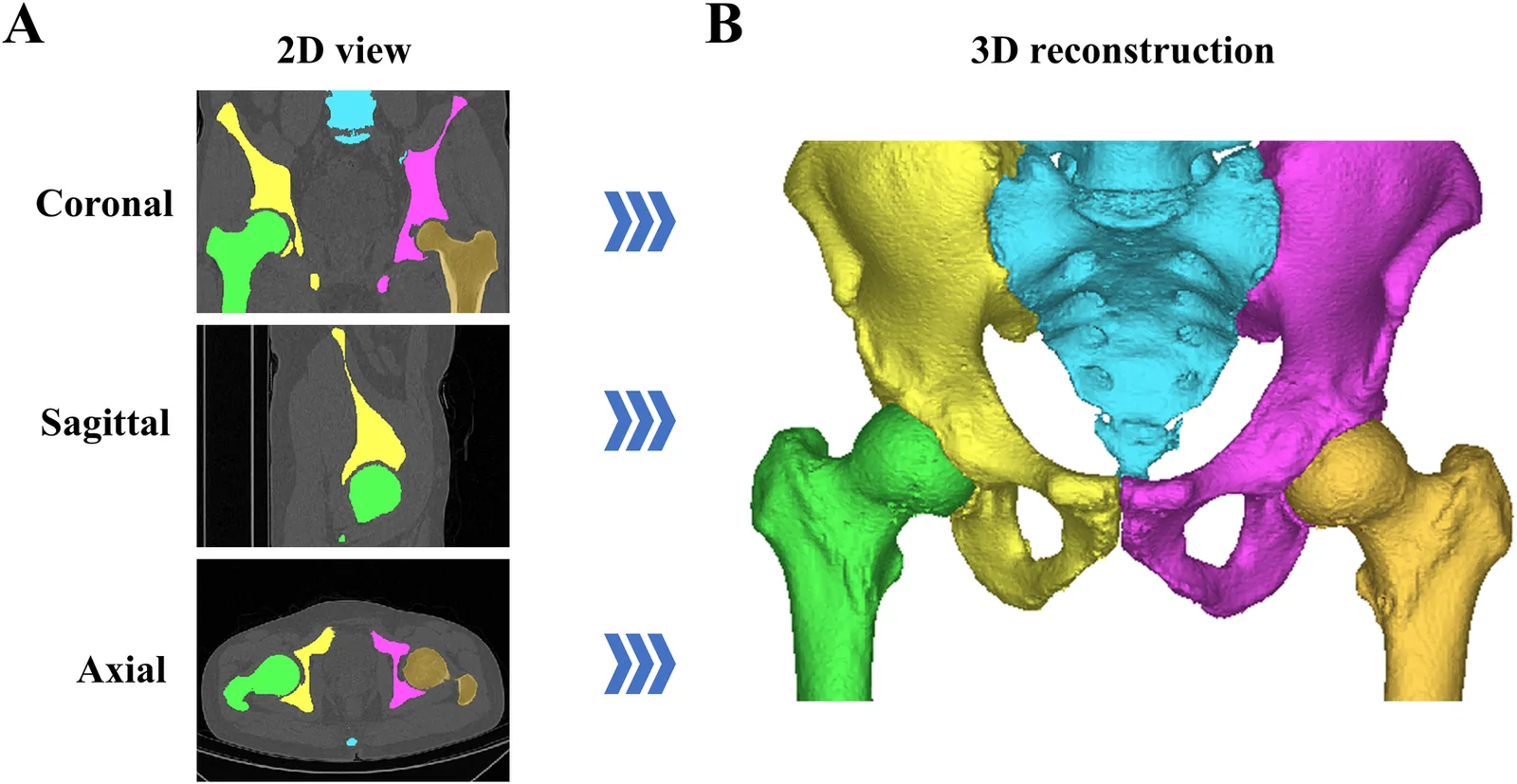
Three-dimensional CT reconstruction images. For each patient, personalized processing was performed using Mimics software to conduct reconstruction of CT images to obtain a 3D CT model. A: representative 2D image of hip in coronal, sagittal, and axial plane; B: an established 3D-CT model of hip.

3D-CT Spherical Fitting measurement for Cam Assessment: The 3D models were imported into 3-matic 13.0 (Materialise, Belgium). The “Wave Brush Mark” tool selected normal femoral head regions for spherical fitting, creating a virtual sphere optimally matching the femoral head. Cam deformity was defined as osteophytes protruding beyond this sphere, with the sphere's boundary marking the resection margin (26) (Figure 2). In order to analyze the repeatability and reliability of the spherical fitting calculation, each same procedure was conducted by three different researchers (inter-observer), and each researcher repeated three times (intra-observer).

**Figure 2:**
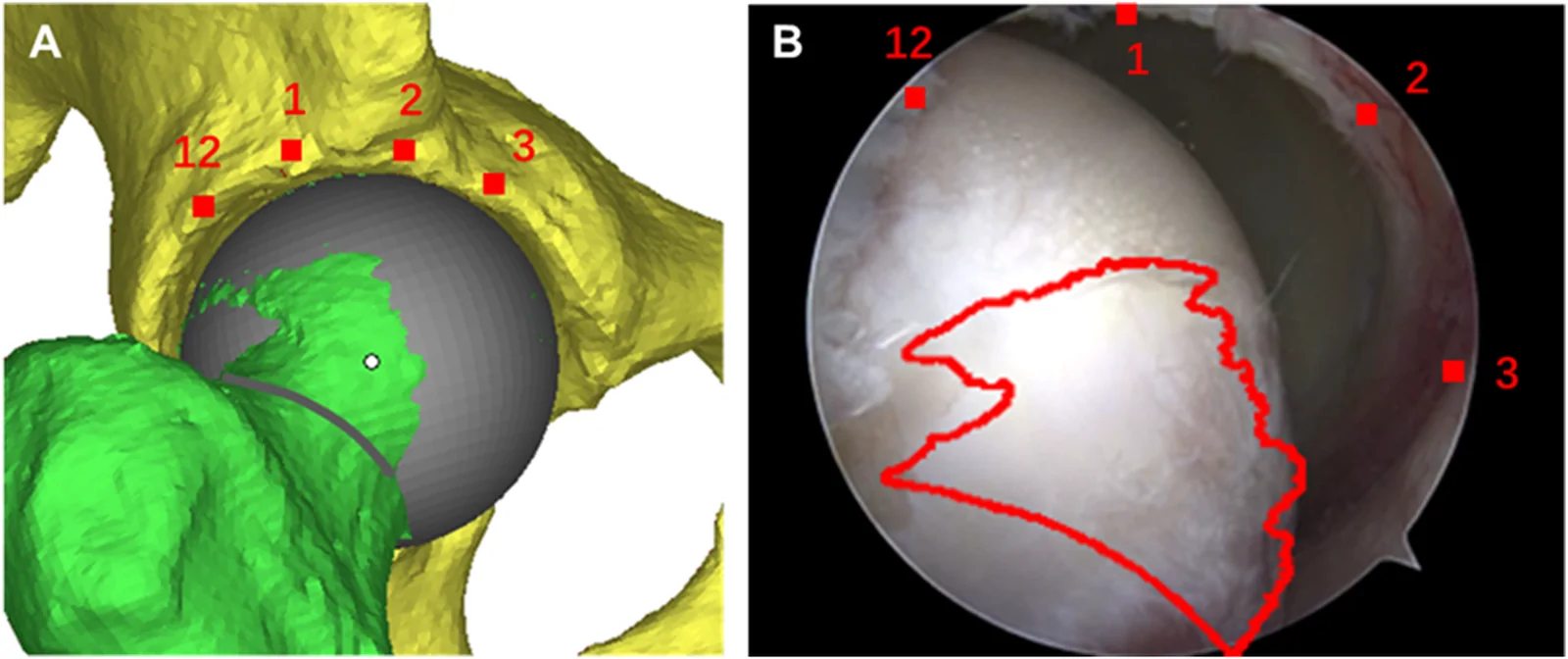
**(A)** method for obtaining virtual spheres in preoperative planning. Using 3-matic software, spherical fitting measurement were performed on CT images to obtain a virtual sphere that best matched the femoral head. The osteophytes protruding beyond the sphere at the femoral head-neck junction were identified as Cam deformity. **(B)** Method for determining surgical resection range through spherical fitting calculation. In preoperative planning, the location of Cam deformity and its arthroscopic resection range were defined using acetabular clock-face positioning.

### 2.3 Patient positioning and surgical approach

Anesthesia: General anesthesia with muscle relaxants. Positioning: Supine on a traction table, with the surgical limb in 15° internal rotation, 0° flexion/extension, and 0° abduction/adduction. The contralateral limb was positioned in 0° rotation, 0° flexion/extension, and 45° abduction. All surgical procedures were performed by a single senior surgeon and all surgical steps were standardized in this study.

Portal Establishment (Outside-in Technique): Anatomical landmarks (anterior inferior iliac spine, greater trochanter) were marked. Three standard portals were established: Anterolateral (AL) Portal: 1 cm anterior and proximal to the greater trochanter. Anterior (AP) Portal: 5 cm distal and 1 cm lateral to the anterior inferior iliac spine. Distal Anterolateral (DALA) Portal: 5 cm distal and 1 cm anterior to the greater trochanter. An extracapsular space was created anteriorly, followed by capsulotomy parallel to the rectus femoris.

### 2.4 Surgical procedures

Acetabular Labral Repair: Longitudinal traction (1 cm joint distraction) allowed entry into the central compartment. Torn labra were repaired using 1–2 suture anchors at the acetabular rim (27). Femoroplasty: A 4.0-mm burr resected Cam osteophytes according to preoperative plans. We used dynamic examination by moving the hip from extension into flexion, 45° abduction, internal rotation and external rotation while directly visualizing the motion with the arthroscope. These sequential maneuvers allow for a dynamic assessment of the Cam deformity and the region requiring resection. Capsular Closure: The capsule was closed with 2–3 nonabsorbable sutures in an interrupted or “lasso-loop” configuration (28).

### 2.5 Postoperative rehabilitation

Day 1: Isometric exercises to restore neuromuscular control and hip muscle strength (e.g., abductors, quadriceps). Weeks 2–4: Gradual weight-bearing with crutches; full weight-bearing by week 4. Range of Motion: Continuous passive motion (CPM) machine for hip/knee mobility. Hip external rotation and abduction were limited to <20°, and flexion to <90° for 4 weeks.

### 2.6 Outcome measures

All patients underwent unilateral hip MRI and CT within one week after surgery to assess whether the *α* angle and the femoral head-neck eccentricity had returned to normal (*α* angle < 50°, femoral head-neck eccentricity < 0.17). Additionally, three-dimensional reconstruction and spherical fitting calculations were performed again on the postoperative CT scans to evaluate whether the Cam deformity had been completely corrected.

The follow-up protocol required all participants to return for scheduled visits at 3, and 6 months post-surgery. The same surgeon conducted the follow-up assessments, and all data were collected prospectively using standardized case report forms. No patients withdrew or were lost to follow-up; thus, the dataset contained no missing values for the primary or secondary outcomes. Data integrity was ensured by independent double-checking of all entries against source documents. Any incomplete forms would have been resolved by immediate review with the attending surgeon, but this situation did not occur. By comparing and analyzing the changes in the modified Harris Hip Score (mHHS) and the Visual Analogue Score (VAS) for pain before and after surgery. This was done to evaluate the therapeutic outcomes of patients after undergoing hip arthroscopic surgery.

### 2.7 Statistical analysis

Statistical analysis was performed using SPSS 22.0 (SPSS Inc., USA). Continuous variables [including age, body mass index (BMI), *α* angle, femoral head-neck offset, mHHS, and VAS] followed a normal distribution as confirmed by the Shapiro–Wilk test and were expressed as mean ± standard deviation. Intergroup comparisons were conducted using independent samples t-tests. For intra-group comparisons (before and after surgery), the *α* angle and femoral head-neck offset were analyzed using paired t-tests, while mHHS and VAS were compared via analysis of variance (ANOVA). Statistical differences between time points were tested, followed by *post hoc* multiple comparisons to assess whether postoperative time points differed significantly from preoperative values. Nonparametric data (e.g., gender) were presented as frequencies (cases, %), and intergroup comparisons were performed using the Pearson chi-square test. Interobserver reliability measurements were calculated using the Shrout-Fleiss intraclass correlation coefficient (ICC) for the assessment of the alpha angle, head-neck offset ratio and spherical fitting results. An interpretive scale for ICC values (< 0.40, poor; 0.4–0.59, fair; 0.60–0.74, good; 0.75–1.00, excellent) was chosen. Statistical significance was regarded as *p* < 0.05. The significance level was set at *α* = 0.05 (two-tailed).

## 3 Results

29 patients with Cam-type FAI met the inclusion criteria, including 12 females and 17 males, in which 13 patients were left side and 16 patients were right side. The mean age was 35.8 ± 13.4 years (range: 22–51). Mean BMI was 22.6 ± 3.3 kg/m² (Table 1).

**Table 1:** Patient characteristics.

Items	Value
Cases	29
Age, y	35.8 ± 13.4 (22–51)
Gender (female)	12 (41.38%)
Gender (male)	17 (58.62%)
Left side	13 (44.83%)
Right side	16 (55.17%)
Body mass index	22.6 ± 3.3 kg/m²
Preoperative alpha angle	75.3 ± 6.3 degrees
Preoperative head–neck offset ratio	0.16 ± 0.02
Acetabular labral condition	Labral tear

All patients exhibited typical FAI symptoms and signs that persisted for over 6 months despite conservative treatment. Preoperative MRI confirmed an alpha angle >55°, femoral head-neck offset ratio <0.17, and acetabular labral tears in all cases, meeting the diagnostic criteria for Cam-type FAI and indicating eligibility for hip arthroscopy.

Preoperatively, 3D-CT spherical fitting measurement enabled precise localization of Cam deformities and optimized osteophyte resection planning. The intra-observer ICC of the spherical fitting measurement was 0.96 (95% CI: 0.91–0.99), and the inter-observer ICC was 0.94 (95% CI: 0.87–0.98), indicating excellent reliability. The Outside-in hip arthroscopy technique simplified portal establishment by eliminating the need for traction and fluoroscopy. Combined with longitudinal capsulotomy, the Outside-in technique provided enhanced arthroscopic visualization, fully exposing Cam deformities and labral tears. All arthroscopic procedures-including labral repair, femoroplasty, and capsular closure-were completed successfully.

Postoperatively, 3D-CT spherical fitting confirmed complete Cam resection in all patients (Figure 3). Postoperative MRI demonstrated satisfactory labral repair. Interobserver reliabilities of the alpha angle, head-neck offset ratio and postoperative spherical fitting completeness showed good to excellent (ICC distributed from 0.754 to 0.891). The mean alpha angle significantly decreased from 75.3 ± 6.3 degrees preoperatively to 44.2 ± 4.1 degrees postoperatively (*p* = 0.017). The femoral head-neck offset ratio elevated from 0.16 ± 0.02 preoperatively to 0.20 ± 0.01 postoperatively (*p* = 0.033) (Figure 4).

**Figure 3:**
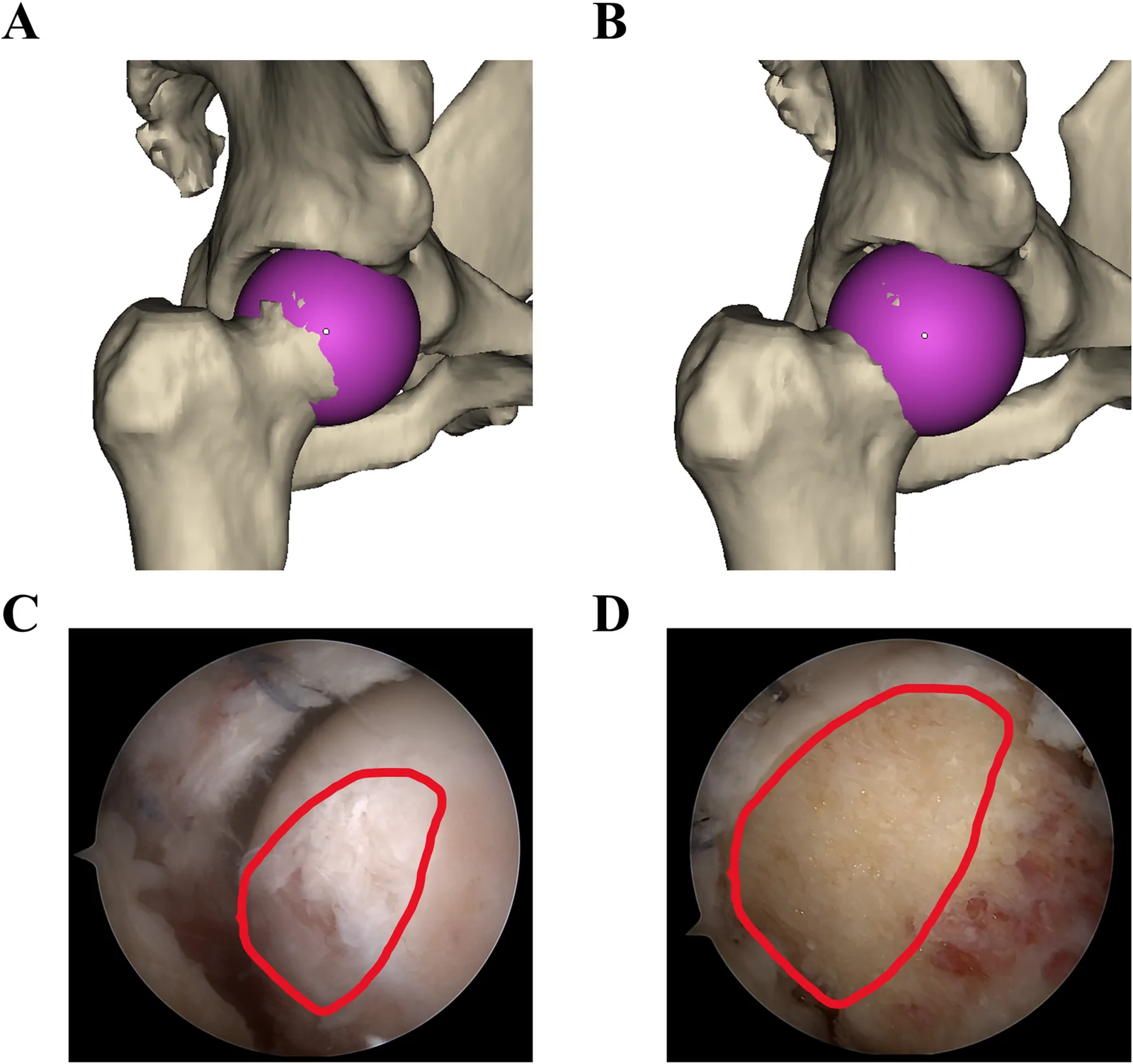
Changes of Cam deformity in 3D-CT and arthroscopic view between pre- and post-operation. **(A)** The Cam deformity in 3D-CT was shown by spherical fitting calculation of femoral head pre-operatively. **(B)** The Cam deformity was corrected entirely in 3D-CT. **(C)** The Cam deformity was located in arthroscopic view (red circle). **(D)** The arthroscopic resection range of Cam deformity after the femoroplasty.

**Figure 4:**
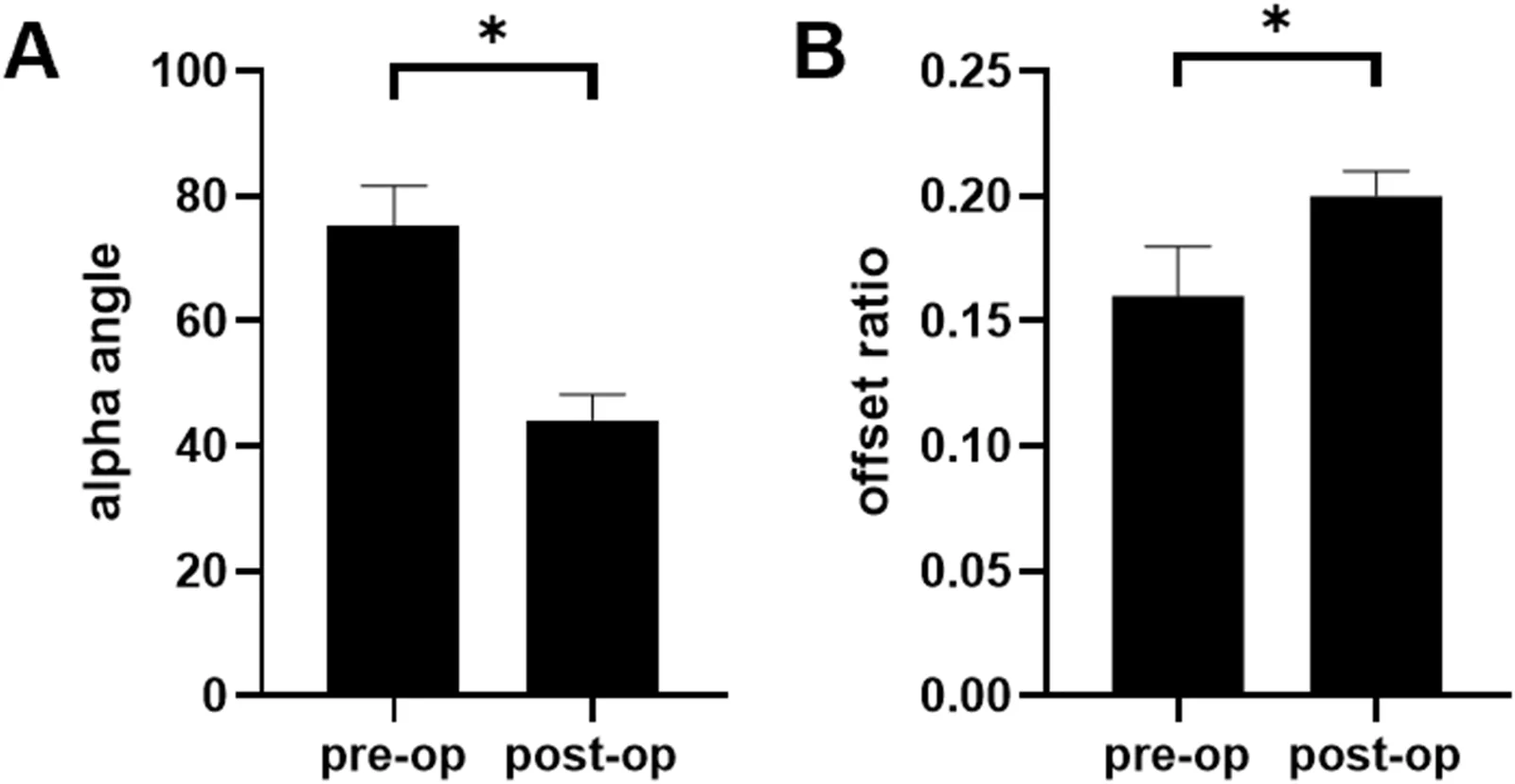
Changes in alpha angle **(A)** and femoral head-neck offset ratio **(B)** between pre- and post-operation. The mean alpha angle significantly decreased from 75.3 ± 6.3 degrees preoperatively to 44.2 ± 4.1 degrees postoperatively (*p* = 0.017). The femoral head-neck offset ratio elevated from 0.16 ± 0.02 preoperatively to 0.20 ± 0.01 postoperatively (*p* = 0.033).

The cohort was followed no loss. No complications (e.g., infection, neurapraxia, joint instability, or revision surgery) were observed. The average mHHS increased sequentially from 52.5 ± 11.7 to 3 months (73.2 ± 14.5, *p* = 0.015) and 6 months (77.9 ± 15.8, *p* = 0.010) after surgery. The average VAS decreased significantly from 3.4 ± 0.9 to (2.4 ± 0.6, *p* = 0.029) at 3 months and (2.3 ± 0.8, *p* = 0.025) at 6 months after surgery (Figure 5).

**Figure 5:**
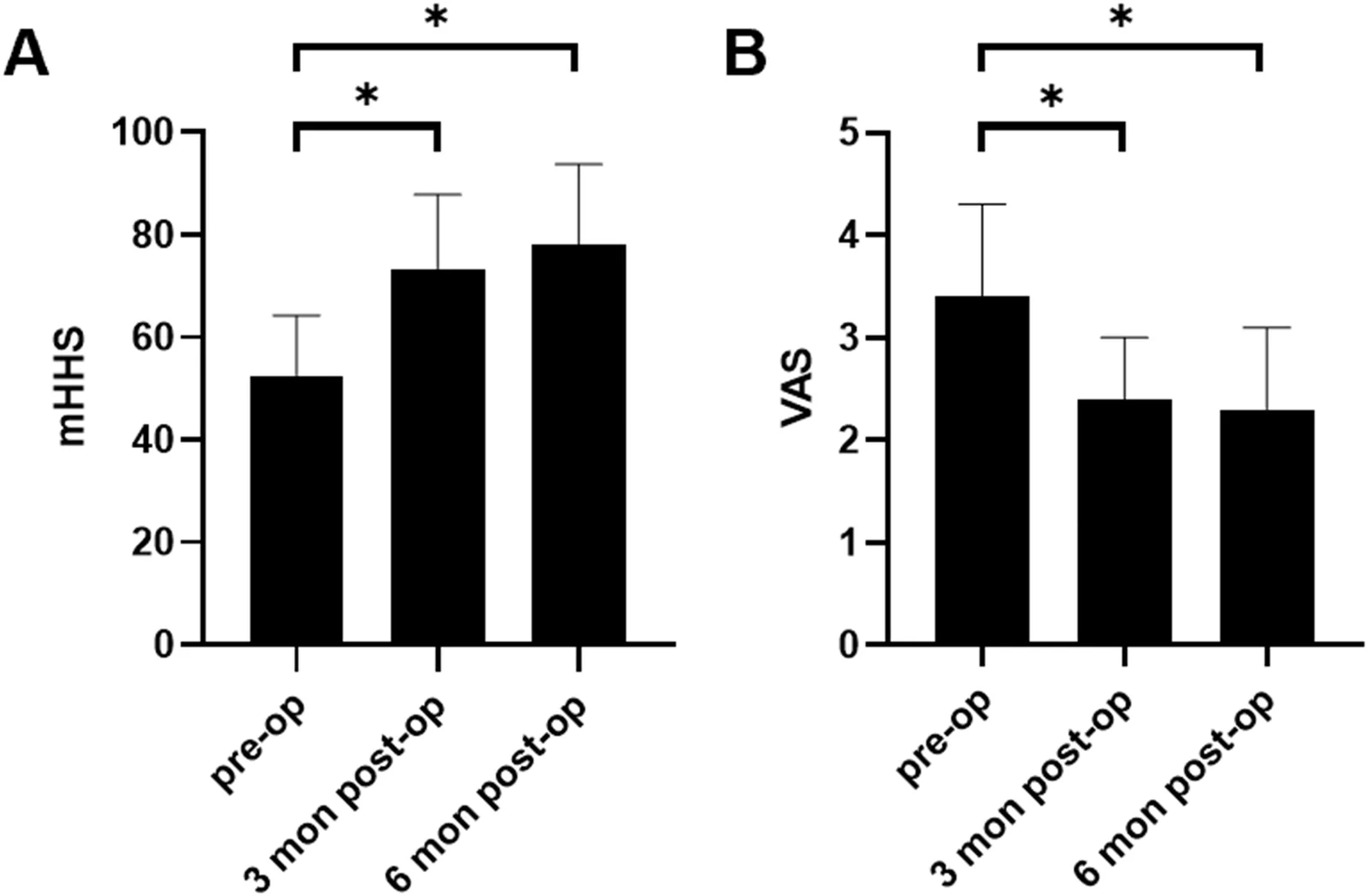
Changes in mHHS **(A)** and VAS **(B)** between pre- and post-operation. The average mHHS increased sequentially from 52.5 ± 11.7 to 3 months (73.2 ± 14.5, *p* = 0.015) and 6 months (77.9 ± 15.8, *p* = 0.010) after surgery. The average VAS decreased significantly from 3.4 ± 0.9 to (2.4 ± 0.6, *p* = 0.029) at 3 months and (2.3 ± 0.8, *p* = 0.025) at 6 months after surgery.

## 4 Discussion

This study is the first to apply 3D-CT spherical fitting technology to preoperative planning for Cam deformity. By creating a virtual sphere that optimally matches the femoral head, the boundaries of Cam deformity protruding beyond the sphere were precisely marked. Results demonstrated that this technique enabled accurate preoperative planning of Cam resection. Postoperative imaging confirmed complete Cam resection in all patients, with significant improvements in clinical functional scores.

Current clinical evaluation of Cam deformity relies on combined imaging modalities (x-ray, CT, MRI) (26, 29, 30). However, imaging remains insufficient for surgical guidance, as it fails to provide precise 3D quantification of osteophyte size and extent. While x-ray is convenient, it lacks comprehensive 3D spatial information. MRI can offer superior accuracy over x-ray (31) and detects osteophytes at any femoral head-neck junction location, but cannot assess overall hip joint biomechanics (32). 3D-CT is widely used for preoperative planning (33), but its utility is limited by subjective interpretation.

The information provided by CT 3D reconstruction is still insufficient for surgeons in diagnosis and treatment. Surgeons can only make simple judgments based on their own experience through observation of the 3D-CT, and the results are usually not accurate enough to accurately determine the specific position and boundary range of Cam deformity in the 3D model, especially the upper bound of Cam deformity (8, 13, 34). Therefore, it is impossible to accurately plan the surgical resection region.

The alpha angle introduced by Nötzli et al. is the primary imaging parameter for Cam assessment (35). Measured on MRI oblique axial slices, it represents the angle between the femoral head center, the point where the femoral head-neck contour deviates from a best-fit circle, and the narrowest femoral neck midpoint. A higher alpha angle indicates a more superior Cam boundary, while multiple abnormal alpha angles across slices suggest broader anterior-posterior Cam extent (9, 10). Postoperative success is defined by an alpha angle <50° and femoral head-neck offset ratio <0.17, which correlate with improved outcomes (23, 25). These thresholds were adopted in this study to evaluate Cam correction. The results of this study showed that postoperative alpha angle was 44.2 ± 4.1 degrees, significantly lower than that (75.3 ± 6.3 degrees) preoperatively. The results were similar with previously reported results (23, 24), which proved the feasibility and effectiveness of the spherical fitting technique.

This study integrated 3D-CT spherical fitting into preoperative planning to restore the femoral head's spherical structure (36). Using MIMICS software (Materialise, Belgium), CT scans (slice thickness <2 mm) were reconstructed into 3D models. A virtual sphere was fitted to the femoral head's normal regions, with osteophytes protruding beyond the sphere defining Cam boundaries. Comparative studies show that spherical fitting outperforms alpha angle-based methods in delineating Cam extent (19). The spherical fitting technique in this study can not only locate the extent of Cam deformity, but also show the resection range and depth of Cam deformity. It can help surgeons to achieve precise resection intraoperatively.

By transforming the boundary of Cam deformity from subjective empirical judgment into objective three-dimensional quantitative indicators, this technique is expected to reduce the variability in surgical planning among surgeons with different levels of experience and improve the homogeneity of surgical outcomes. This technique can be integrated into preoperative planning, intraoperative decision-making, and postoperative evaluation for hip arthroscopy, particularly in patients with femoroacetabular impingement or other hip deformities. As for the target patient population, this technique is most beneficial for patients with complex Cam lesions, particularly moderate-to-severe deformities, post-traumatic femoral head irregularities, or revision cases where conventional imaging is insufficient.

In terms of potential cost and time of the 3D-CT spherical fitting technique, this technique requires thin-slice CT scanning (slice thickness <2 mm), specialized 3D reconstruction software (MIMICS), and additional preoperative planning time (approximately 30–60 min based on our center's experience). This may increase initial healthcare resource investment and collaboration costs between the radiology and orthopedics departments. However, from a health economics perspective, if more precise resection can reduce the rate of revision surgery due to residual bone or over-resection and delay the time to joint replacement, the long-term benefits may outweigh the short-term costs.

The Outside-in hip arthroscopy technique was first proposed by Dienst et al. in 2005 and has been validated through clinical practice as a feasible method for establishing surgical portals (25, 31). This technique replaces the traditional Inside-out approach, which uses the Seldinger percutaneous puncture method to create arthroscopic portals from inside to outside. In recent years, studies on the Outside-in technique have increased, all reporting satisfactory surgical outcomes (23, 32–36).

With the assistance of the Outside-in technique, surgeons efficiently completed arthroscopic procedures. Postoperative mHHS and VAS were significantly improved compared to preoperative values (*p* < 0.05). Clinical functional scores progressively increased over time, with no complications such as infection, neurapraxia, hip instability, or revision surgery observed.

Outside-in technique has some advantages in hip arthroscopy. It has simplified operative workflow. No specialized arthroscopic instruments are required, making the procedure easier to learn and more beginner-friendly. Meanwhile, it is radiation-free. Eliminates the need for C-arm fluoroscopy, reducing radiation exposure for both surgeons and patients. Besides, it is superior for complex cases. Particularly effective for patients with severe Pincer deformity or hip stiffness, where traditional Inside-out techniques struggle to establish access. Nevertheless, the current study had several limitations. The retrospective design with a small sample size is may introduce limited statistical power and bias of the outcomes, future studies with larger sample sizes and multi-center designs are needed. This study lacks a control group (standard arthroscopy without spherical fitting technique), which results in the inability to demonstrate the advantages of spherical fitting technique compared to traditional method. All surgeries were performed by a single senior surgeon, which means that the prognosis may rely on surgeons. Longer-term validation is necessary and a detailed analysis using minimal clinical important difference is needed (37, 38).

## 5 Conclusion

With the assistance of the 3D-CT spherical fitting technique and the outside-in hip arthroscopy technique, surgeons can accurately evaluate the Cam deformity and plan the excision range of osteophyte preoperatively and perform accurate resection of Cam deformity during the surgery. This technique transforms the Cam deformity from subjective empirical judgment into objective 3D quantitative indicators, reducing variability among surgeons with different experience levels and improving the homogeneity of surgical outcomes. It is applicable to preoperative planning, intraoperative decision-making, and postoperative evaluation for hip arthroscopy, and is particularly beneficial for complex Cam lesions. From a health economics perspective, if it can reduce revision surgeries and delay the time to joint replacement.

## Data Availability

The raw data supporting the conclusions of this article will be made available by the authors, without undue reservation.
